# Association between impairment and self-rated health: a brazilian population study considering type, origin, and degree of limitation

**DOI:** 10.1186/s12889-023-15445-w

**Published:** 2023-03-28

**Authors:** Paula Anderle, Patrícia Klarmann Ziegelmann, Bárbara Niegia Garcia de Goulart

**Affiliations:** Universidade Federal do Rio Grande do Sul Programa de Pós-graduação em Epidemiologia, Rua Ramiro Barcelos 2400, CEP 90035-003 Porto Alegre, Brazil

**Keywords:** Impaired persons, Self-Assessment, Health surveys, Epidemiology

## Abstract

**Background:**

Perceived health is a well-known, low-cost measure in public health, and has been used in several studies on individuals with impairment. Although many studies have related impairment to self-rated health (SRH), few have considered the origin and degree of limitation of the impairment. This study examined whether physical, hearing, or visual impairments—when analyzed according to origin (congenital or acquired) and degree of limitation (with or without)—are associated with the SRH status.

**Methods:**

This cross-sectional study used data of 43,681 adult individuals from the Brazilian National Health Survey (NHS, 2013). The outcome SRH was dichotomized into poor (including the regular, poor, and very poor responses) or good (including the good and very good responses). Crude and adjusted (for socio-demographic characteristics and chronic diseases history) prevalence ratios (PR) estimates were evaluated using Poisson regression models with the robust variance estimator.

**Results:**

Poor SRH prevalence was estimated at 31.8% (95%CI:31.0–33.0) among the non-impaired population, 65.6% (95%CI:60.6–70.0) among individuals with physical impairment, 50.3% (95%CI:45.0–56.0) for people with hearing impairment, and 55.3% (95%CI:51.8–59.0) for the visually impaired. Individuals with congenital physical impairment—with or without limitations—presented the strongest association with the poorest SRH status. Participants with non-limiting, congenital hearing impairment showed a protective factor to poor SRH (PR = 0.40 95%CI: 0.38–0.52). Individuals with acquired visual impairment with limitations demonstrated the strongest association with poor SRH (PR = 1.48 95%CI:1.47–1.49). Among the impaired population, middle-aged participants showed a stronger association with poor SRH than older adult participants.

**Conclusions:**

Impairment is associated with poor SRH status, especially among people with physical impairment. The origin and degree of limitation of each type of impairment differently impacts SRH among the impaired population.

**Supplementary Information:**

The online version contains supplementary material available at 10.1186/s12889-023-15445-w.

## Background

Currently, approximately 15% of the world’s population suffers from some form of impairment or considerable functional difficulties [1, 2]. Data from the Brazilian National Health Survey (NHS, 2013) show that 6.2% of the Brazilian population has some type of impairment, with vision and hearing loss affecting 3.6% and 1.1% of the population, respectively. These sensory problems severely limit the daily activities of 16% and 21% of people with vision and hearing impairments, respectively; physical impairment affects more than 2.6 million Brazilians (approximately 1.3% of the population), with 46.8% of these individuals experiencing intense or extremely intense limitations in daily activities [3].

In this study, we use the terms *disability* and *impairment* according to the World Health Organization (WHO) definitions: disability is an umbrella term for impairments, activity limitation, and participation restriction. It denotes the negative aspects of the interaction between an individual (with a health condition) and his/her contextual factors (environmental and personal factors). Impairments are problems in body function and structure, such as a significant deviation or loss [4].

Measurement of the health status of several populations has been performed using what is known as self-rated health (SRH)—which is essentially a single question: “How do you evaluate your health?” Respondents are given a scale to classify their health as very good, good, regular, poor, or very poor. SRH is a low-cost, easy-to-apply tool that considers the contextual frameworks of an individual’s health status, such as cultural and psychosocial aspects, social participation, health behaviors, and lifestyle [5]. Despite its non-specific nature, studies have shown that SRH and objective health measures are associated, suggesting that SRH is a significant predictor of mortality as well as chronic diseases, impairment, and functional decline [1, 6–11], thus highlighting its significance as a construct to measure health status.

Investigations regarding the health status of populations with impairment using the SRH tool have been gaining space in the national and international literature. Visually impaired, hearing impaired, and physically impaired individuals has been found associated with poor SRH [12–16]. Another study showed that visual impairment indirectly predicted mortality with SRH as a mediator [17]. However, none of those studies analyzed the origin and degree of limitation.

In this context, a systematic review has demonstrated that individuals with any type of acquired impairment and a high degree of limitation due to this impairment have worse SRH compared to the general population [11, 18, 19]. Additionally, aging in this population increases the association with poor SRH status [20].

Studies on congenital and acquired impairments have argued that an individual’s behavior and self-concept of impairment, as well as their age and time of exposure to the impairment, can influence the SRH status. Thus, people with congenital impairments, and who have adaptive behaviors, are associated with better SRH compared to those with impairment acquired later in life. Furthermore, aging in this population increases the association with poor SRH status [16, 21, 22].

Meanwhile, very few studies have focused on middle-aged populations with impairments. A few authors have argued that more demanding work activities can lead to limited physical functions. In this population, promoting self-care and preventing physical decline can improve health status and reduce poor SRH scores among older individuals [23, 24].

Given this scenario, the research hypothesis of this study is that the SRH status among individuals with physical or sensory impairments varies based on origin (congenital or acquired) and limitation (i.e., whether daily activities are limited).

We believe that classifying SRH into these strata can facilitate clarifying epidemiological phenomena, such as the social determinants of health, participation in social activities, and how these individuals use health services. The data collected against this local backdrop can lend support to public health promotion and prevention campaigns that better target these populations and their heterogeneous needs.

Thus, this study aims to assess whether physical, hearing, or vision impairment, when analyzed according to origin (congenital or acquired) and degree of limitation (with or without), are associated with the SRH status.

## Methods

### Study Design and Data Sourcing

This is a cross-sectional study conducted using the data from the Brazilian NHS collected in 2013 by the Brazilian Institute of Geography and Statistics (IBGE), in partnership with the Ministry of Health and the Oswaldo Cruz Foundation (Fiocruz) [25]. Structured into 20 modules, the NHS database is representative of the Brazilian population addressing socio-demographic aspects, use of public and private health services, health status, and lifestyle. The study was approved by the National Research Ethics Commission under number 10853812.7.0000.0008.

### Sampling

The NHS is a household survey with probabilistic sampling aimed at estimating the Brazilian population. It was developed as a Master Sample of the IBGE’s Integrated Home Survey System. Cluster sampling was used and the stratification of the Primary Sampling Units (PSU)—that is, geographic regions—was divided into three stages: (i) PSU census sectors; (ii) PSU households; and (iii) an adult resident respondent (aged 18 years or more) per household. Households and residents were selected via simple random sampling [25, 26]. Details about the sampling plan are presented in a published article [26]. In total, 64,348 households were selected, and 60,202 interviews were conducted.

### Self-rated health

The SRH status—that is, the outcome of this study—was obtained through self-reports. The participants answered the question: “In general, how would you assess your health: very good, good, regular, poor, or very poor?” For the analyses, the SRH outcome was dichotomized into poor (including the regular, poor, and very poor responses) or good (including the good and very good responses) to facilitate interpretation. Dichotomizing the variables to include the regular response in the bad category made the conclusions more conservative than a three-category classification (in which the regular response was a separate category). Sensitivity analysis considering two other scenarios of how to categorize the SRH outcome was performed. Scenario 2 is the binarization of SRH into poor (very poor + poor + regular) and not poor (very good + good); and scenario 3 is considering SRH into three categories: poor (very poor + poor), regular and not poor (very good + good).

### Exposure factors

Three exposure factors were considered: physical impairment, hearing impairment, and visual impairment (also called sensory impairments) measured through self-reports. These factors were analyzed in two ways:

1. As a binary factor (having or not having impairment): individuals who answered positively to the question “Do you have a physical impairment?” were considered impaired. For sensory impairments, those who answered positively to the questions: “Do you have a hearing impairment?” or “Do you have a visual impairment?” were considered impaired.
2. As a factor with five categories: no impairment, a congenital impairment without limitations, a congenital impairment with limitations, an acquired impairment without limitations, and an acquired impairment with limitations. To measure limitation, the following question was asked: “To what extent does this impairment limit your usual activities?” To answer, respondents were given a 5-point Likert Scale. In this study, limitations were categorized as “limited” (i.e., moderately limited, very limited, and very intensely limited) and “minimally limited/not limited.” As for the question about the origin of their impairment, the participants answered the following question: “Were you born with this impairment or was it acquired?” (answered as born or acquired).

### Adjustment factors

For adjustment, socio-demographic variables were used: sex (male or female), age (considered quantitatively), skin color (categorized as white and non-white; among the non-whites, there were black, brown, indigenous, and other minority ethnicities’ participants), education (categorized as elementary, secondary, undergraduate, graduate) and employment (paid or not; paid activity was understood as work in the formal or informal labor markets resulting in monetary gain). Additionally, a health variable for chronic diseases was also included, namely Diabetes mellitus, cardiovascular disease (including hypertension, heart disease, and stroke), lung disease, cancer, arthritis, and depression. This variable was constructed using answers to questions such as: “Have you ever had a medical diagnosis of ___?,” to which the possible answers were “yes” or “no.” Thus, the variable of chronic diseases was categorized into: no disease, one disease, two diseases, and three or more diseases.

### Data Analysis

The population studied were described using absolute and relative frequencies. Owing to the complexity of the sample, the relative frequencies and all other results were weighted (the sampling characteristics, non-responses, and calibration). To evaluate association between impairment and SRH, Poisson regression models with robust variance estimation were adjusted. For each type of impairment, two models were considered: one with the impairment as a binary factor, and another with impairment as a five-categories factor [27, 28]. As such, by considering non-impairment as the reference category, crude and adjusted prevalence ratios were estimated. Adjustment factors are described above and enter into the model even if they were nor significant (p > 0.05). For each model, interaction between impairment and age as a binary factor (< 60 or > = 60 years old) was assessed and the results presented. Association results are presented as weighted prevalence ratios with 95% confidence interval, which were calculated using the survey and sandwich packages in R software [29].

## Results

A total of 60,202 individuals, aged 18 years old or older, responded to the Brazilian NHS survey. From these, we excluded 578 participants who had more than one impairment; a population of 59,624 remained. Of these, 9,958 did not answer questions regarding education and ethnicity and 7,637 did not answer questions about chronic disease. These participants were also excluded from the study, resulting in a final sample of 43,681 participants.

The distributions of impairment and SRH did not differ between study and excluded populations (p = 0.52 and p = 0.71). Additionally, there was no significant difference between the prevalence of poor SRH comparing the included and excluded populations for all populations (no impairment, physical impairment, and hearing impairment), except for the visual impairment population (Table 1; supplementary material).

The study sample (43,681 individuals) had an average age of 44.20 years (SD = 15.73). The prevalence of physical impairment was estimated at 1.44% (95% CI: 1.25–2.00), hearing impairment at 1.77% (95% CI: 1.57–2.00), and visual impairment at 4.70% (95% CI: 4.32–5.00).

We used the SRH outcome in its binary format (poor or not poor). Comparing with the SRH in its five categories original format (very poor, poor, regular, good, and very good), we observed that the “poor” category of the binary version predominantly comprised the “regular” response (40.7%, 42.3%, and 41.0% for physical, hearing, and visual disabilities, respectively and 28.2% for no disability); the “not poor” category predominantly comprised the “good” responses (28.2%, 34.3%, and 37.8% for physical, hearing, and visual disabilities, respectively and 52.7% for no disability) regardless of the group—whether impaired or not (Fig. 1; supplementary material). Sensitivity analysis regarding the way to categorize the SRH outcome showed that the way we used it in this study results in the lowest strength of association between impairment and SRH.

Poor SRH prevalence was estimated at 31.8% (95%CI:31.0–33.0) among the non-impaired population, and at 65.6% (95%CI:60.6–70.0) among individuals with physical impairment, 50.3% (95%CI:45.0–56.0) among individuals with hearing impairment, and 55.3% (95%CI:51.8–59.0) among those with visual impairments. Furthermore, for the three types of impairment, there are certain imbalances regarding the socio-demographic characteristics and chronic diseases between the groups of impaired participants and the group of people with no impairments (Table 1). Fewer individuals with impairments hold a graduation degree compared to those without impairments (10.6% vs. 18.0%), but have more elementary education than their counterparts without impairments (65.0% vc 42.3%); they are also less employed (46.0% vs. 60.8%). The socio-demographic characteristics and chronic diseases of the excluded population are presented in the supplementary material.

**Table 1:** Distribution of socio-demographic characteristics, self-rated health status, and chronic diseases according to the type of impairment of the study participants—National Health Survey, 2013. *

	Total Population n = 43,681	Physical Impairment n = 613		Hearing Impairment n = 637		Visual Impairment n = 2,083	No Impairment n = 40,248	
	**n(%)** ^w*^	**n(%)** ^w*^		**n(%)** ^w*^		**n(%)** ^w*^	**n(%)** ^w*^	
**Total**	43,681 (100.0)	613 (1.4)		737 (1.7)		2,083 (4.7)	40,248 (92.0)	
**Self-rated health**								
Good	28,596 (66.3)	195 (34.4)		333(49.7)		944 (44.7)	27,124 (68.2)	
Poor	15,085 (33.7)	418 (65.6)		404(50.3)		1,139 (55.3)	13,124 (31.8)	
**Sex**								
Male	17,675 (45.0)	368 (59.0)		354(49.7)		808 (41.8)	16,145 (44.8)	
Female	26,006 (55.0)	245 (41.0)		383(50.3)		1,275 (58.2)	24,103 (55.2)	
**Age (years)**								
18–29	8,537 (21.0)	39 (6.4)		42 (6.2)		133 (7.1)	8,323 (22.2)	
30–39	10,799 (22.7)	99 (16.0)		73 (9.0)		214 (9.5)	10,413 (23.8)	
40–49	9,075 (20.0)	137 (17.4)		116(14.8)		415(18.5)	8,407 (20.2)	
50–59	7,241 (18.1)	141 (27.0)		144(19.0)		537(26.4)	6,419 (17.5)	
60 or more	8,029 (18.2)	197 (33.2)		362(51.0)		784(38.5)	6,686 (16.3)	
**Skin color**								
Non-white	25,090 (50.2 )	378 (57.3)		346(37.2)		1,138 (46.3)	23,228 (50.1)	
White	18,591 (49.8)	235 (42.7)		391(62.8)		945 (53.7)	17,020 (49.9)	
**Education**								
Elementary	19,161 (44.0)	385 (65.0)		475 (63.8)		1,272 (62.1)	17,029 (42.3)	
Secondary	16,360 (37.8)	154 (24.5)		169 (24.7)		526 (27.0)	15,511 (38.8)	
Graduate	7,680 (17.3)	69 (10.2)		87(10.6)		272 (10.3)	7,252 (18.0)	
Post-Graduate	480 (0.8)	5 (0.3)		6 (0.7)		13 (0.6)	456 (0.9)	
**Employment**								
Yes	25,384 (59.3)	188 (30.0)		285 (39.0)		935 (46.4)	23,976 (60.8)	
No	18,297 (40.7)	425(70.0)		452 (66.3)		1,148 (53.6)	16,272 (39.2)	
Chronic disease**								
No	28,657 (64.4)	247 (41.8)		296(39.5)		924 (44.3)	27,190 (66.3)	
One	3,814 (9.0)	87 (16.2)		80(11.3)		254 (12.0)	3,393 (8.5)	
Two	6,992 (16.3)	145 (22.0)		196(27.8)		467 (20.7)	6,184 (15.8)	
Three or more	4,218 (10.3)	134 (20.0)		165(21.4)		438 (23.0)	3,481 (9.4)	

* All analyses were performed using weighted data that included sampling characteristics, non-responses and calibration. Data was expanded to the Brazilian population

** Cardiovascular diseases, lung disease, cancer, diabetes mellitus, depression and arthritis

The prevalence of physical impairment was estimated at 1.4% and distributed into: 1.10% (95% CI: 0.96–1.10) “acquired with limitations”; 0.10% (95% CI: 0.06–0.15) “congenital with limitations”; 0.40% (95% CI: 0.31–1.00) “acquired without limitations”; and 0.20% (95% CI: 0.07–0.20) “congenital without limitations” (Table 1; supplementary material). Adjusted analyses showed a more significant association (p < 0.001) between physical impairment and poor SRH status compared to no impairment and SRH status. Significant interactions with age were found. Participants under 60 showed a stronger association with poor SRH status (PR = 1.52, 95%CI 1.49–1.56) when compared with older adults. The same occurred when physical impairment was categorized. Congenitally impaired adults (≥ 60 years) with no limitations showed a strong association with poor SRH reports (PR = 2.12, 95%CI 1.96–2.29). The other categories also presented significant, albeit weaker, associations (Table 2). No significant interactions were found between the others adjustment factors and physical impairment (results not shown).

### Hearing impairment

The prevalence of hearing impairment was estimated at 1.7%, with 0.62% (95% CI: 0.55–1.00) attributed to losses “acquired with limitations,” 0.06% (95% CI: 0.03–0.09) to “congenital with limitations,” 1.60% (95% CI: 1.45–2.00) to “acquired without limitations,” and 0.13% (95% CI: 0.09–0.20) to “congenital without limitations” (Table 2; supplementary material). Adjusted analyses showed a significant association (p < 0.001) between hearing impairment and poor SRH reports when compared with no hearing loss and SRH status. The congenital category with no limitations showed a protection factor against poor SRH status (PR = 0.40, 95%CI 0.39–0.43). Significant interactions with age were found. Participants under 60 showed a stronger association with poor SRH reports (PR = 1.16, 95%CI 1.13–1.19) than older adults. The same occurred when hearing impairment was categorized. Among older adults (≥ 60 years), both categories of congenital impairment showed a protection factor against poor SRH status. No significant interactions were found between the other adjustment factors and the hearing impairment factor (results not shown).

### Visual impairment

The prevalence of visual impairment was estimated at 4.7% and distributed into: 1.32% (95% CI: 1.20–2.00) “acquired with limitations”; 0.20% (95% CI: 0.11–0.25) “congenital with limitations”; 3.70% (95% CI: 3.30–4.00) “acquired without limitations”; and 0.36% (95% CI: 0.27–0.48) “congenital without limitations” (Table 3; supplementary material). Adjusted analyses showed a more significant association (p < 0.001) between visual impairment and poor SRH reports compared to no impairment and SRH status. Significant interactions with age were identified. Participants under 60 showed a stronger association with poor SRH status (PR = 1.37; 95%CI 1.35–1.39) than older adults. The same occurred when visual impairment was categorized. The categories with limitations showed the strongest associations with poor SRH reports for both acquired (PR = 1.61; 95% CI: 1.59–1.62) and congenital (PR = 1.42; 95% CI: 1.38–1.45) impairments. Adults with acquired impairments (≥ 60 years) and limitations showed a strong association with poor SRH status (PR = 1.37; 95% CI: 1.35–1.38); acquired impairments with no limitations showed no significant association with poor SRH status (Table 2). No significant interactions were identified between the other adjustment factors and physical impairment (results not shown).

**Table 2:** Crude and adjusted analyses *(Poisson regression) of impairments in relation to poor self-rated health status—National Health Survey, 2013

Poor Self-Rated Health Status					
	WP (CI 95%)	PR_crude_ (CI95%)	PR_adj_ (CI 95%)	PR_adj_ (CI 95%)	PR_adj_ (CI 95%)
				≥ 60 years old	< 60 years old
**Physical impairment**					
Yes	65.58 (64.91–62.25)	2.06 (2.03–2.09)^a^	1.42 (1.39–1.44)^a^	1.27(1.25–1.30)^a^	1.52(1.49–1.56)^a^
No	33.25 (33.17–33.32)	1	1	1	1
**Physical impairment**					
Acquired with limitations	73.03 (72.24–73.83)	2.29 (2.26–2.33)^a^	1.44 (1.40–1.47)^a^	1.27(1.23–1.30)^a^	1.62(1.56–1.68)^a^
Congenital with limitations	66.30 (64.33–68.24)	2.08 (1.99–2.17)^a^	1.58 (1.54–1.61)^a^	1.30(1.29–1.32)^a^	1.62(1.56–1.67)^a^
Acquired without limitations	48.70 (47.33-50.00)	1.53 (1.44–1.62)^a^	1.24 (1.22–1.27)^a^	1.03(1.01–1.05)^a^	1.30(1.27–1.32)^a^
Congenital without limitations	54.16 (51.69–56.62)	1.70 (1.56–1.85)^a^	1.58 (1.49–1.67)^a^	2.12(1.96–2.29)^a^	1.34(1.29–1.40)^a^
No	33.25 (33.17–33.32)	1	1	1	1
**Hearing impairment**					
Yes	50.32 (49.76–50.88)	1.58 (1.54–1.62)^a^	1.09 (1.06–1.11)^a^	1.10(1.06–1.14)^a^	1.16(1.13–1.19)^a^
No	31.80 (31.71–31.87)	1	1	1	1
**Hearing impairment**					
Acquired with limitations	53.98 (52.60-55.37)	1.69 (1.61–1.78)^a^	1.05 (1.01–1.09)^a^	0.98(0.94–1.04)	1.33(1.27–1.40) ^a^
Congenital with limitations	33.88 (33.00-34.77)	1.06 (0.98–1.15)	1.06 (0.98–1.14)	0.80(0.78–0.82)^a^	1.07(0.98–1.16)
Acquired without limitations	53.41 (52.76–54.06)	1.68 (1.64–1.72)^a^	1.14 (1.11–1.18)^a^	1.17(1.11–1.23)^a^	1.21(1.17–1.25) ^a^
Congenital without limitations	10.62 (10.51–10.73)	0.33 (0.30–0.36) ^a^	0.40 (0.39–0.43) ^a^	0.44(0.38–0.52) ^a^	0.39(0.38–0.41) ^a^
No	31.80 (31.71–31.87)	1	1	1	1
**Visual impairment**					
Yes	55.30 (54.92–55.68)	1.74 (1.71–1.76)^a^	1.22 (1.20–1.23)^a^	1.10(1.07–1.12)^a^	1.37(1.35–1.39)^a^
No	31.80 (31.71–31.87)	1	1	1	1
**Visual impairment**					
Acquired with limitations	78.10 (77.84–78.35)	2.45 (2.43–2.47)^a^	1.48 (1.47–1.49)^a^ 1.46 (1.44–1.48)^a^	1.37(1.35–1.38)^a^	1.61(1.59–1.62)^a^
Congenital with limitations	70.38 (68.83–71.93)	2.21 (2.14–2.28)^a^	1.11 (1.08–1.13)^a^	1.31(1.30–1.32)^a^	1.42(1.38–1.45)^a^
Acquired without limitations	48.50 (48.01–48.98)	1.52 (1.49–1.55)^a^	1.21 (1.14–1.27)^a^	0.99(0.96–1.03)	1.27(1.23–1.31)^a^
Congenital without limitations	42.00 (39.76–44.04)	1.31 (1.16–1.48)^a^	1	1.21(1.19–1.23)^a^	1.23(1.11–1.37)^a^
No	31.80 (31.71–31.87)	1		1	1

*Adjustment factors: socio-demographic characteristics (age, sex, skin color, education, and employment) and chronic disease (cardiovascular diseases, lung disease, cancer, diabetes mellitus, depression, and arthritis)

^a^ p-value < 0.001

WP: weighted prevalence. PRcrude: crude prevalence ratio. PRadj: adjusted prevalence ratio

## Discussion

In this study, both physical and sensory (hearing and visual) impairments were found to be significantly associated with poor health perception. Poor SRH prevalence was estimated at 31.8% (95%CI:31.0–33.0) among the non-impaired population; 65.6% (95%CI:60.6–70.0) for persons with physical impairment; 50.3% (95%CI:45.0–56.0) for individuals with hearing impairment; and 55.3% (95%CI:51.8–59.0) for those with visual impairments.

Our results concur with those of other studies that indicated that individuals with impairments tend to report a poorer SRH compared to individuals with no impairments [11, 30]. Those associations were found even after adjustments for socio-demographic and health variables. The adjustment factors we used are well-documented in the literature and are related to individuals’ SRH [20, 31]. Given the nature of this study’s methodology (cross-sectional survey), we cannot confirm that impairment causes poor SRH; however, the identified association highlights a significant issue to consider in public health planning policies and actions worldwide.

Furthermore, the associations between impairment and SHR differ based on age; younger people with impairments are more likely to rate their health as poor compared to older people. In general, individuals below age 60 with physical impairments—acquired or congenital—had poorer SRH status compared to their seniors. The literature shows that individuals with physical impairments constitute the largest unattended subpopulation, presenting a 75% higher chance of having unmet health needs, not only related to physical architectural barriers [30], but associated to social stigmas that people with physical impairments and limitations have to manage while trying to access public and private environments. For example, access to public services, transportation from home to healthcare facilities, prejudice and ignorance about the needs of regular life and health that physically impaired people need, etc. These aspects may justify the greater association with poor SRH status found in our study in this population. Moreover, because they are in a socially and economically active age-group, individuals under 60 years of age are more socially and physically active, and therefore, more exposed to social and architectural hindrances. A novel finding of our study was that the association between each type of impairment and poor SRH depends on the origin (congenital or acquired) and degree of limitation caused by the impairment.

In this study, visual acquired impairments in older aadults with limitations showed strong association with poor SRH, probably because lack of vision is associated with loss of autonomy in daily life activities. Additionally, adults below age 60 with physical impairments showed a stronger association with poor SRH compared to their older counterparts. Subjects with congenital physical impairments with no limitations and aged 60 or more showed a strong association with poor SRH. Even though adjustments by age and clinical history were made in this study, probably there are other associated factors that lead to poor SRH in physically impaired individuals—even those with congenital physical impairments and no limitations. Further studies are required to investigate this association.

Individuals with congenital hearing impairment with limitations were not associated with poor SRH; congenital hearing impairment with no limitations seemed to be a protective factor against poor SRH. This can be explained by the fact that congenital hearing impaired individuals develop other mechanisms to communicate than speaking and hearing. Additionally, after access to hearing aids and/or rehabilitation, they develop coping strategies to integrate with the community, such as sign language, orofacial reading, etc. Moreover, hearing impairment is a type of “silent impairment” that does not impede physical mobility and integration into the community, even though, at times, spoken messages may not be understood [22, 32].

Similarly, it is believed that people with congenital impairments that do not limit daily activities are capable of developing optimal functionality [33]. They social participation ensures a better quality of life and, consequently, a higher perception of health. This is different from the experience of those who have lost crucial functions and require rehabilitation to resume their activities. Furthermore, a systematic review indicated that motivational factors influenced by cognitive, emotional, and social aspects encourage social participation and a better quality of life [34]—these associated factors influence the SRH status.

Several studies have indicated the relationship between poor SRH status and older disabled populations [35–37]. These studies have mainly defined disability as limitation of body functions and daily activities due to illness or injury [38]. In our research, the term *impairment* was used to define changes in body functions caused by structural deterioration and restriction [38]. A significant association between poor SRH status and middle-aged participants with all the impairments studied, highlights the significance of further research on this population [24]. We hypothesize that middle-aged people are more economically and socially active; therefore, they experience situations of discrimination and limitation more frequently because of their impairment, which leads to poor SRH status [39].

Additionally, it is significant to consider the conceptual model that guides this study [40], and certain points that must be highlighted. As multiple factors influence the perception of health among populations with some form of impairment, such as sociodemographic aspects, disease, social participation, and limited daily activities, attention must be paid to the dynamics of these factors in these groups. When the three types of impairment were analyzed, the population characteristics sketched a profile of unemployed, non-white individuals with low educational attainment and chronic diseases. The influence of social determinants on health should be considered when studying impaired populations. The dynamics of each type of impairment should also be examined—physical or sensory, acquired or congenital. Environments of social vulnerability and unfavorable socioeconomic status can contribute to a poor perception of health. Thus, the results presented here must be weighed together with these factors, as they contribute to a poorer perception of health among these groups. Such careful analyses can guide the use of public resources when creating public health strategies and policies.

This study considered the origin (congenital or acquired) and degree of limitation caused by the impairment. Additionally, the dataset used is from a representative sample of the Brazilian adult population in which health status was measured through an SRH tool. SRH assessments are widely used in health surveys worldwide, and are significant predictors of mortality [5, 6, 9], morbidity [7, 8], and use of health services [11, 30], in addition to being directly related to sociodemographic indicators such as sex, age, and education [31]. The SRH status has been compared to objective measures of health [2] and recommended by the World Health Organization (WHO) and the Centers for Disease Control and Prevention (CDC) [41], as a reliable measure of population health as well as a relevant tool for mapping population health status and facilitating the management of health policies and actions.

Since 2002, Brazil has been following the National Health Policy for People with Disabilities, which is focused on the inclusion of people with disabilities in the entire service network of the Unified Health System (SUS) and recognizes the need to implement the process of responses to the complex issues that involve healthcare for people with disabilities in Brazil. Therefore, this study uses actualized data of the Brazilian disabled population according to age range, origin, and grade of self-perceived limitation and self-rated heath. It can serve as a guide to other middle-income countries with similar demographic characteristics to plan policies for disease prevention and health promotion.

In closing, we believe that this study has filled a gap in the investigations regarding the SRH status of impaired populations, according to type, origin, and degree of limitation. This type of research is still in infancy; however, it is highly relevant in the current domestic as well global scenarios because it addressed a crucial theme associated with health policies of nations.

Moreover, as demographic profile worldwide have been changing, disabilities and impairments have been increasing. Therefore, population-based studies are necessary because they facilitate simultaneous evaluation of health scenarios of specific countries, and enable comparison of different populations worldwide. Furthermore, the data were given a weighted analysis and yielded results that can be extrapolated to the entire Brazilian population.

Additionally, information about the impairments was obtained through self-reports and depended on the personal understanding of each participant and their values, conception of health, and culture [5, 22]. In this regard, a review study indicated the significance of standardized definitions of impairments, as there is considerable variation in self-reports about impairments during census [42]. Other limitations should be mentioned: (a) when performing the age-stratification analysis, we indicated that the congenital category with limitations had a small number of participants over 60 years old, and this may have led to an overestimation of the association with the worst perception of health; this should be taken in consideration; and (b) the Brazilian NHS does not provide complete information about the use of assistive devices for the impairments we addressed. This information is crucial for analysis because the use of assistive devices by impaired participants can address limitations to daily activities and the self-perception of health. Thus, we draw attention to the need to include this type of information in the NHS and in future studies that examine the use of assistive devices among impaired populations that perform self-perceived health assessments.

## Conclusion

In Brazil, physical and sensory impairments are associated with poor SRH, especially among adults below 60 years old. These association depend on the following factors: type of impairment (physical, hearing, or visual); whether congenital or acquired impairment; and whether impairment causes limitations. Among individuals with physical and visual impairments, acquired conditions that result in limitations were the strongest categories associated with poor SRH for both age categories—that is, below and above 60 years old. Regarding hearing impairment, associations differ: congenital without limitations presented as a protective factor to poor SRH; acquired—whether with or without limitation—presented as a risk factor. Our results suggest the significance of stratifying impairments to better understand their dynamic influence on SRH.

## Electronic supplementary material

Below is the link to the electronic supplementary material.

Supplementary Material 1

Supplementary Material 2

## Data Availability

Data are available on the Ministry of Health website, and can be accessed through the link below: http://www.saude.gov.br/vigilancia-em-saude/indicadores-de-saude/pesquisa-nacional-de-saude-pns.

## List of abbreviations

NHS: National Health Survey
CI: Confidence interval
SRH: Self-rated health
IBGE: Brazilian Institute of Geography and Statistics
FIOCRUZ: Oswaldo Cruz Institute Foundation
PSU: Primary Sampling Units
SD: Standard Deviation
CDC: Center for Disease Control and Prevention
