# Supplementary material for: Association between impairment and self-rated health: a brazilian population study considering type, origin, and degree of limitation
Source: BMC Public Health. 2023 Mar 28;23:580. doi: 10.1186/s12889-023-15445-w (PMC10045530; doi:10.1186/s12889-023-15445-w)
Supplement: Supplementary file 1 — Supplementary Material 1 [file 12889_2023_15445_MOESM1_ESM.doc]

SUPPLEMENTARY MATERIAL:

Table 1: Distribution of socio-demographic characteristics, self-rated health, and chronic diseases according to the origin and limitation of physical disability of the study participants – National Health Survey, 2013. *

| **Physical impairment** | | | | | |
| --- | --- | --- | --- | --- | --- |
|  | **No**  **Impairment**  **n=40,248**  **n(%)^w*^** | **Acquired with**  **limitation**  **n=374**  **n(%)^w*^** | **Congenital with**  **limitation**  **n=43**  **n(%)^w*^** | **Acquired without**  **limitation**  **n=152**  **n(%)^w*^** | **Congenital without**  **limitation**  **n=44**  **n(%)^w*^** |
| **Self-rated health** |  |  |  |  |  |
| Good | 27,124 (68.2) | 85 (27.0) | 9 (33.7) | 72 (48.7) | 21 (45.8) |
| Poor | 13,124 (31.8) | 289 (73.0) | 34 (66.3) | 80 (51.3) | 23 (54.2) |
| **Sex** |  |  |  |  |  |
| Male | 16,145 (44.8) | 225 (59.3) | 23 (38.2) | 99 (71.8) | 21 (33.6) |
| Female | 24,103 (55.2) | 149 (40.7) | 20 (61.8) | 53 (28.2) | 23 (66.4) |
| **Age (years)** |  |  |  |  |  |
| 18-29 | 8,323 (22.2) | 11 (3.3) | 6 (14.6) | 14 (6.8) | 8 (24.0) |
| 30-39 | 10,413 (23.8) | 44 (8.6) | 11 (35.8) | 28 (26.0) | 16 (31.3) |
| 40-49 | 8,407 (20.2) | 84 (17.7) | 12 (18.0) | 34 (20.6) | 7 (5.0) |
| 50-59 | 6,419 (17.5) | 89 (30.3) | 8 (19.6) | 38 (23.8) | 6 (14.2) |
| 60 or more | 6,686 (16.3) | 146 (40.1) | 6 (12.0) | 38 (22.8) | 7 (25.5) |
| **Skin color** |  |  |  |  |  |
| Not White | 23,228 (50.1) | 227 (59.9) | 32 (77.8) | 94 (50.5) | 25 (40.1) |
| White | 17,020 (49.9) | 147 (40.1) | 11 (22.2) | 58 (49.5) | 19 (59.9) |
| **Education** |  |  |  |  |  |
| Elementary School | 17,029 (42.3) | 225 (70.0) | 26 (60.8) | 82 (59.2) | 22 (43.5) |
| High School | 15,511 (38.8) | 78 (22.1) | 13 (20.2) | 47 (31.2) | 16 (27.5) |
| Graduation | 7,252 (18.0) | 36 (7.3) | 4 (19.0) | 23 (9.6) | 6 (29.0) |
| Post-Graduation | 456 (0.9) | 5 (0.6) | - | - | - |
| **Employment** |  |  |  |  |  |
| Yes | 23,976 (60.8) | 82 (21.2) | 12 (38.8) | 67 (37.8) | 27 (73.4) |
| No | 16,272 (39.2) | 292 (78.7) | 31 (61.2) | 85 (62.2) | 17 (26.6) |
| **Chronic disease**** |  |  |  |  |  |
| No | 27,190 (66.3) | 122 (36.0) | 25 (60.1) | 73 (51.8) | 27 (44.1) |
| One | 3,393 (8.5) | 56 (14.7) | 3 (8.1) | 23 (20.8) | 5 (21.8) |
| Two | 6,184 (15.8) | 99 (25.6) | 6 (12.3) | 33 (18.4) | 7 (11.1) |
| Three or more | 3,481 (9.4) | 97 (23.7) | 9 (19.5) | 23 (9.0) | 5 (23.0) |

* All analyses were performed using weighted data that considered the sampling characteristics, non-response and calibration. Data expanded for the Brazilian population.

** Cardiovascular diseases, lung disease, cancer, Diabetes Melittus, depression and arthritis.

Table 2: Distribution of socio-demographic characteristics, self-rated health, and chronic diseases according to the origin and limitation of hearing impairment of the study participants – National Health Survey, 2013. *

| **Hearing Impairment** | | | | | |
| --- | --- | --- | --- | --- | --- |
|  | **No**  **Impairment**  **n=40,248**  **n(%)^w*^** | **Acquired with**  **limitation**  **n=187**  **n(%)^w*^** | **Congenital with**  **limitation**  **n=25**  **n(%)^w*^** | **Acquired without**  **limitation**  **n=478**  **n(%)^w*^** | **Congenital without**  **limitation**  **n=47**  **n(%)^w*^** |
| **Self-rated health** |  |  |  |  |  |
| Good | 27,124 (68.2) | 56 (46.0) | 10 (33.8) | 228 (46.6) | 13 (10.6) |
| Poor | 13,124 (31.8) | 131 (54.0) | 15 (66.2) | 250 (53.4) | 34 (89.4) |
| **Sex** |  |  |  |  |  |
| Male | 16,145 (44.8) | 82 (47.0) | 7 (30.0) | 247 (52.2) | 18 (29.0) |
| Female | 24,103 (55.2) | 105 (53.0) | 18 (70.0) | 231 (48.8) | 29 (71.0) |
| **Age (years)** |  |  |  |  |  |
| 18-29 | 8,323 (22.2) | 3 (1.1) | 6 (20.2) | 21 (5.0) | 12 (34.5) |
| 30-39 | 10,413 (23.8) | 11 (6.8) | 7 (33.2) | 43 (8.0) | 12 (20.7) |
| 40-49 | 8,407 (20.2) | 23 (12.7) | 2 (21.8) | 84(15.2) | 7 (18.0) |
| 50-59 | 6,419 (17.5) | 31 (14.6) | 6 (18.9) | 98 (20.5) | 9 (17.6) |
| 60 or more | 6,686 (16.3) | 119 (64.7) | 4 (5.9) | 232 (51.3) | 7 (9.1) |
| **Skin color** |  |  |  |  |  |
| Not White | 23,228 (50.1) | 85 (33.0) | 15 (64/0) | 222 (39.0) | 24 (27.0) |
| White | 17,020 (49.9) | 102 (67.0) | 10 (36.0) | 256 (61.0) | 23 (73.0) |
| **Education** |  |  |  |  |  |
| Elementary School | 17,029 (42.3) | 134 (71.7) | 13 (36.7) | 301 (66.0) | 18 (19.9) |
| High School | 15,511 (38.8) | 41 (24.0) | 6 (24.2) | 105 (22.8) | 17 (48.7) |
| Graduation | 7,252 (18.0) | 12 (4.3) | 6 (39.0) | 57 (10.0) | 12 (31.4) |
| Post-Graduation | 456 (0.9) | - | - | 6 (1.2) | - |
| **Employment** |  |  |  |  |  |
| Yes | 23,976 (60.8) | 42 (28.2) | 7 (28.9) | 206 (39.5) | 30 (81.2) |
| No | 16,272 (39.2) | 145 (71.8) | 18 (71.1) | 272 (60.5) | 17 (18.8) |
| **Chronic disease**** |  |  |  |  |  |
| No | 27,190 (66.3) | 66 (30.5) | 12 (52.5) | 186 (40.5) | 32 (60.8) |
| One | 3,393 (8.5) | 22 (12.4) | 6 (36.1) | 51 (11.0) | 1 (0.5) |
| Two | 6,184 (15.8) | 52 (31.2) | 5 (7.9) | 134 (27.5) | 5 (24.6) |
| Three or more | 3,481 (9.4) | 47 (25.9) | 2 (3.7) | 107 (21.0) | 9 (14.1) |

* All analyses were performed using weighted data that considered the sampling characteristics, non-response and calibration. Data expanded for the Brazilian population.

** Cardiovascular diseases, lung disease, cancer, Diabetes Melittus, depression and arthritis.

Table 3: Distribution of socio-demographic characteristics, self-rated health, and chronic diseases according to the origin and limitation of visual impairment of the study participants – National Health Survey, 2013. *

| **Visual disability** | | | | | |
| --- | --- | --- | --- | --- | --- |
|  | **No**  **Disability**  **n=40,248**  **n(%)^w*^** | **Acquired with**  **limitation**  **n=481**  **n(%)^w*^** | **Congenital with**  **limitation**  **n=61**  **n(%)^w*^** | **Acquired without limitation**  **n=1,438**  **n(%)^w*^** | **Congenital without**  **limitation**  **n=103**  **n(%)^w*^** |
| **Self-rated health** |  |  |  |  |  |
| Good | 27,124 (68.2) | 118 (21.9) | 21 (29.6) | 696 (48.5) | 63 (58.0) |
| Poor | 13,124 (31.8) | 363 (78.1) | 40 (70.4) | 742 (51.5) | 40 (42.0) |
| **Sex** |  |  |  |  |  |
| Male | 16,145 (44.8) | 193 (39.0) | 22 (41.1) | 551 (42.9) | 42 (39.4) |
| Female | 24,103 (55.2) | 288 (61.0) | 39 (58.9) | 887 (57.1) | 61 (60.6) |
| **Age (years)** |  |  |  |  |  |
| 18-29 | 8,323 (22.2) | 19 (4.2) | 11 (23.0) | 82 (5.9) | 21 (22.3) |
| 30-39 | 10,413 (23.8) | 39 (7.1) | 18 (28.2) | 138 (8.3) | 19 (22.1) |
| 40-49 | 8,407 (20.2) | 87 (16.8) | 15 (13.2) | 293 (19.4) | 20 (18.8) |
| 50-59 | 6,419 (17.5) | 132 (32.0) | 9 (18.0) | 377 (26.1) | 19 (14.7) |
| 60 or more | 6,686 (16.3) | 204 (39.9) | 8 (17.6) | 548 (40.3) | 24 (22.1) |
| **Skin color** |  |  |  |  |  |
| Not White | 23,228 (50.1) | 283 (52.0) | 33 (36.1) | 769 (44.1) | 53 (55.6) |
| White | 17,020 (49.9) | 198 (48.0) | 28 (63.9) | 669 (55.9) | 50 (44.4) |
| **Education** |  |  |  |  |  |
| Elementary School | 17,029 (42.3) | 349 (75.4) | 27 (60.0) | 849 (59.5) | 47 (44.5) |
| High School | 15,511 (38.8) | 95 (19.4) | 21 (19.2) | 375 (28.2) | 35 (44.2) |
| Graduation | 7,252 (18.0) | 36 (4.6) | 11 (19.5) | 205 (11.6) | 20 (10.5) |
| Post-Graduation | 456 (0.9) | 1 (0.5) | 2 (1.3) | 9 (0.7) | 1 (0.8) |
| **Employment** |  |  |  |  |  |
| Yes | 23,976 (60.8) | 153 (34.2) | 28 (30.0) | 695 (49.7) | 60 (54.8) |
| No | 16,272 (39.2) | 328 (65.8) | 34 (70.0) | 743 (50.3) | 43 (45.2) |
| **Chronic disease**** |  |  |  |  |  |
| No | 27,190 (66.3) | 156 (32.8) | 30 (28.4) | 679 (46.6) | 59 (68.0) |
| One | 3,393 (8.5) | 78 (16.0) | 12 (21.0) | 154 (10.5) | 10 (9.3) |
| Two | 6,184 (15.8) | 114 (23.4) | 7 (9.0) | 326 (21.3) | 20 (7.9) |
| Three or more | 3,481 (9.4) | 133 (27.8) | 12 (41.6) | 279 (21.6) | 14 (14.8) |

* All analyses were performed using weighted data that considered the sampling characteristics, non-response and calibration. Data expanded for the Brazilian population.

** Cardiovascular diseases, lung disease, cancer, Diabetes Melittus, depression and arthritis.

|  | **Total population**  **n=15,943** | **Physical impairment**  **n=250** | **Hearing**  **Impairmen**  **n=265t** | | **Visual**  **Impairment**  **n=764** | | **No**  **Impairment**  **N=14,664** |
| --- | --- | --- | --- | --- | --- | --- | --- |
|  | **n(%)^w*^** | **n(%)^w*^** |  | **n(%)^w*^** |  | **n(%)^w*^** | **n(%)^w*^** |
| **Self-rated health** |  |  |  |  |  |  |  |
| Good | 10,412 (67.4) | 85 (33.2) |  | 105 (37.6) |  | 303 (41.9) | 9,919 (69.7) |
| Poor | 5,531 (32.6) | 165 (66.8) |  | 160 (62.4) |  | 461 (58.1) | 4,745 (30.3) |
| **Sex** |  |  |  |  |  |  |  |
| Male | 7,943 (46.7) | 133 (57.7) |  | 148 (59.7) |  | 348 (45.0) | 7,314 (53.5) |
| Female | 8,000 (53.3) | 117 (42.3) |  | 117 (40.3) |  | 416 (55.0) | 7,350 (46.5) |
| **Age (years)** |  |  |  |  |  |  |  |
| 18-29 | 5,776 (41.6) | 31 (10.9) |  | 30 (11.5) |  | 115 (19.0) | 5,600 (43.8) |
| 30-39 | 3,443 (19.1) | 42 (11.3) |  | 32 (10.2) |  | 87 (11.6) | 3,282 (19.8) |
| 40-49 | 2,260 (12.8) | 51 (24.7) |  | 30 (11.8) |  | 103 (12.6) | 2,076 (12.6) |
| 50-59 | 1,693 (10.7) | 52 (25.1) |  | 26 (11.0) |  | 128 (14.2) | 1,487 (10.2) |
| 60 or more | 2,771 (15.8) | 74 (28.0) |  | 147 (55.5) |  | 331 (42.6) | 2,219 (13.6) |
| **Skin color** |  |  |  |  |  |  |  |
| Not White | 10,700 (60.5) | 172 (64.0) |  | 174 (63.7) |  | 491 (57.0) | 9,863 (60.6) |
| White | 5,240 (39.5) | 78 (36.0) |  | 89 (36.3) |  | 273 (43.0) | 4,800 (39.4) |
| **Education** |  |  |  |  |  |  |  |
| Elementary School | 3,439 (55.4) | 45 (72.0) |  | 50 (77.5) |  | 175 (78.0) | 3,169 (54.0) |
| High School | 2,151 (38.4) | 19 (24.6) |  | 13 (20.0) |  | 48 (18.5) | 2,075 (39.6) |
| Graduation | 392 (6.1) | 4 (3.4) |  | 1 (2.5) |  | 10 (3.5) | 377 (6.3) |
| Post-Graduation | 6 (0.1) | - |  | - |  | - | 6 (0.1) |
| **Employment** |  |  |  |  |  |  |  |
| Yes | 8,478 (55.0) | 71 (28.1) |  | 84 (28.5) |  | 282 (41.2) | 8,041 (56.5) |
| No | 7,465 (45.0) | 179 (71.9) |  | 11 (71.5) |  | 482 (58.8) | 6,623 (46.5) |
| **Chronic disease**** |  |  |  |  |  |  |  |
| No | 5,458 (66.4) | 70 (30.8) |  | 73 (32.2) |  | 167 (36.9) | 5,148 (69.6) |
| One | 667 (8.0) | 21 (34.4) |  | 17 (8.6) |  | 62 (14.0) | 567 (7.1) |
| Two | 1,334 (15.6) | 36 (15.8) |  | 55 (31.3) |  | 108 (23.0) | 1,135 (14.8) |
| Three or more | 847 (10.0) | 32 (19.0) |  | 39 (27.9) |  | 121 (26.1) | 655 (8.5) |

Table 4: Distribution of socio-demographic characteristics, self-rated health, and chronic diseases according to the type of disability of the study excluded participants – National Health Survey, 2013. *

* All analyses were performed using weighted data that considered the sampling characteristics, non-response and calibration. Data expanded for the Brazilian population.

** Cardiovascular diseases, lung disease, cancer, Diabetes Melittus, depression and arthritis.

Table 5: Association between impairment and SRH considering 3 scenarios for
categorization of the SRH outcome.

|  | **Scenario 1** | **Scenario 2** | **Scenario 3** |  |
| --- | --- | --- | --- | --- |
|  | **WP(CI95%)** | **WP(CI95%)** | **WPCI95%)** |  |
| **Physical Impairment** |  |  |  |  |
| No | 31.80 1.00 | 4.65 1.00 | 6.38 1.00 |  |
| Yes | 65.58 2.06 (2.03-2.09) | 26.41 5.68 (5.26-6.13) | 43.42 6.81 (6.32-7.33) |  |
| **Hearing Impairment** |  |  |  |  |
| No | 31.80 1.00 | 4.65 1.00 | 6.38 1.00 |  |
| Yes | 50.32 1.58(1.55-1.62) | 8.52 1.83(1.68-1.98) | 14.64 2.29(2.07-2.54) |  |
| **Visual Impairment** |  |  |  |  |
| No | 31.80 1.00 | 4.65 1.00 | 6.38 1.00 |  |
| Yes | 55.30 1.74(1.71-1.76) | 12.61 2.71(2.56-2.88) | 22.00 3.45(3.20-2.71) |  |
|  |  |  |  |  |
|  |  |  |  |  |
|  |  |  |  |  |
|  |  |  |  |  |
|  |  |  |  |  |
|  |  |  |  |  |

Scenario 1: poor (very poor + poor + regular) and not poor (very good + good).
Scenario 2: poor (very poor + poor) and not poor (very good + good + regular).

Scenario 3: poor (very poor + poor), regular and not poor (very good + good).

WP: weighted prevalence.
