# Supplementary material for: Association between impairment and self-rated health: a brazilian population study considering type, origin, and degree of limitation
Source: BMC Public Health. 2023 Mar 28;23:580. doi: 10.1186/s12889-023-15445-w (PMC10045530; doi:10.1186/s12889-023-15445-w)

Figure 1: Distribution of self-rated health open question, according to impairment of the study participants - National Health Survey, 2013.


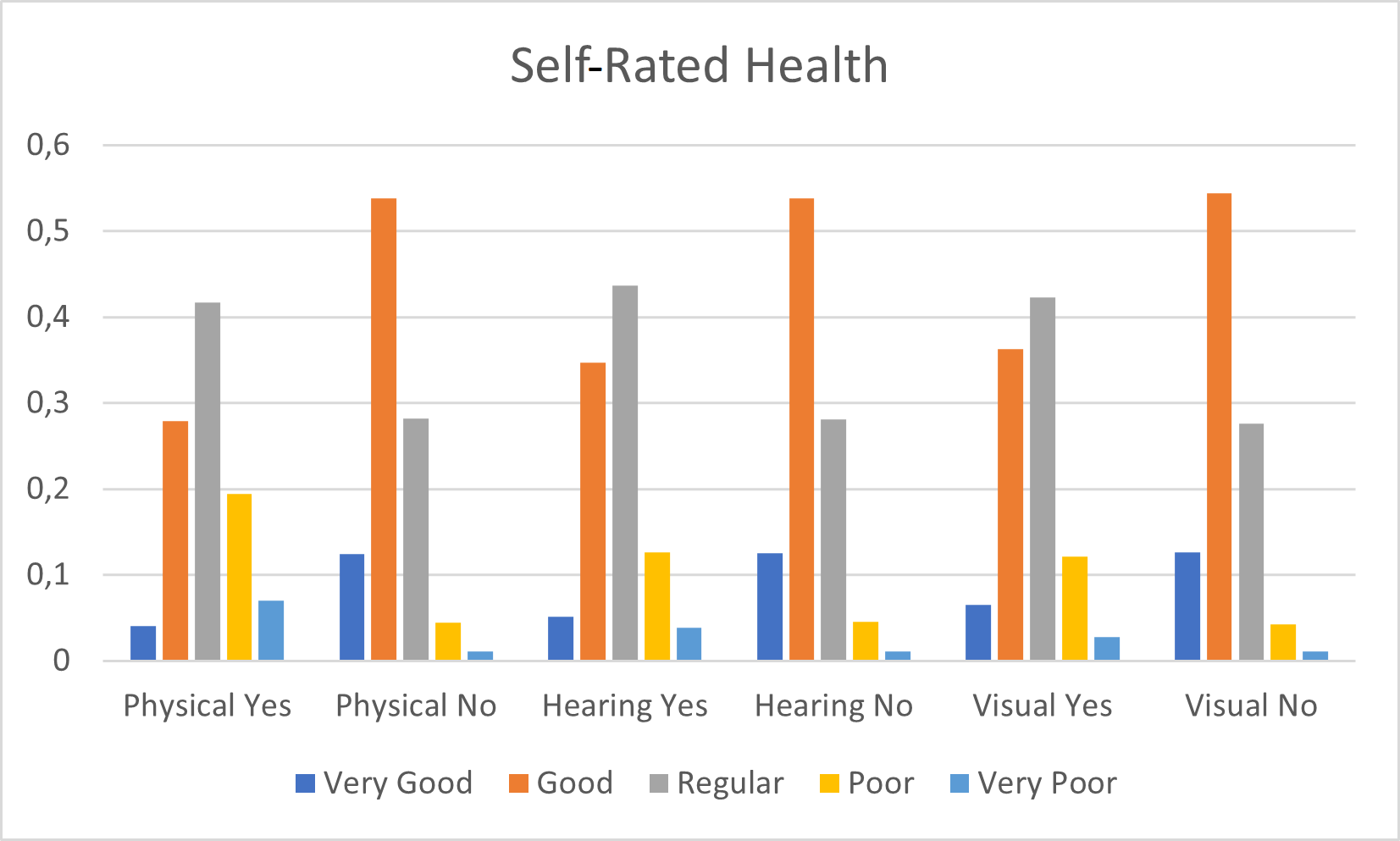

Supplement: Supplementary file 2 — Supplementary Material 2 [file 12889_2023_15445_MOESM2_ESM.doc]
